# Adapting Demirjian Standards for Portuguese and Spanish Children and Adolescents

**DOI:** 10.3390/ijerph191912706

**Published:** 2022-10-04

**Authors:** Lisete S. Mónico, Luís F. Tomás, Inmaculada Tomás, Purificación Varela-Patiño, Benjamin Martin-Biedma

**Affiliations:** 1 Center for Research in Neuropsychology and Cognitive and Behavioral Intervention (CINEICC), Faculty of Psychology and Educational Sciences, Universidade de Coimbra, 3000-115 Coimbra, Portugal; 2 School of Medicine and Dentistry, Health Research Institute Foundation of Santiago (FIDIS), Universidad de Santiago de Compostela, 15703 Santiago de Compostela, Spain

**Keywords:** Demirjian standards, Demirjian method, age estimation, dental age estimation

## Abstract

Estimation of children’s chronological age is highly important in human and forensic sciences. The Demirjian method has been reported as accurate for this purpose. The literature review shows some evidence that the accuracy of estimating chronological age via the Demirjian standards is not a straightforward process. The objective of this research is to analyze the reliability of the Demirjian standards in Portuguese and Spanish children and adolescents and adapt it to include sex and group age as contingent factors. Methods: Orthopantomographs of 574 Portuguese and Spanish male and female children and adolescents were employed to test the reliability of the Demirjian method. After testing for inter-rater consistency and age estimation using the Demirjian standards, multiple regression analysis was performed controlling for sex and age group. Results: The Demirjian standards overestimated chronological age for both sexes, mainly for females. Through the development of regression functions, more detailed dental age estimation was performed. The predictive capacities of the Demirjian method and the significant teeth varied as a function of children’s age. The Demirjian global standard predicted over 65% of the variance of the chronological age. Taking a tooth-by-tooth approach, the predictive ability increased by over 70%. Conclusions: The accuracy of estimating chronological age via the Demirjian method is not as reliable as it might appear, judging from the results found according to age group and according to sex crossed with age group.

## 1. Introduction

Age estimation plays a prominent role in forensic sciences, anthropology, human sciences, medical jurisprudence, pediatrics, and orthodontics, and “(…) is one of the most important characteristics used to establish the identity of any individual in different legal, forensic, or anthropological research context” [1], p. 203. The increase in migratory flow to the European Union countries from the Middle East, Eastern Europe, and Sub-Saharan Africa, along with wars, several mass disasters with destruction or fragmentation of bodies, and also a globalized economy pose legal problems of varying orders (determination of legal adulthood, undocumented persons, random victims, etc.) [2].

The identification of victims is essential not only for humanitarian reasons, but also because it is needed for civil and/or criminal investigations, being used in many different situations such as immigration, child abuse, and criminal examination [3,4,5,6,7]. In the medium or long term, diverse sequelae may arise, psychological as well as social or legal, that may affect relatives and even the general population. Moreover, in mass disasters there is often destruction or fragmentation of bodies associated with high levels of DNA degradation making this identification technique difficult or ineffective, prompting the need to develop other techniques [8,9,10,11,12]. Thus, the questions that are posed to the professionals always go through the identification of the victims in case of catastrophe. A methodology that helps in this identification process nowadays is of crucial importance, increasing the world’s resilience to disasters.

Teething provides valuable information for the identification process through age estimation [7,13,14,15]. It has the great advantage of being less likely to suffer alterations due to nutritional, hormonal, and/or pathological causes [1,16,17,18,19,20]. In cases of road accidents, fires, and other traumatic situations that culminate in death, dentition is often maintained and is the only way to assess the age of the victim. In minors, teething is the best individual and physiological indicator of chronological age [17], and it is also a helpful tool in characterizing missing individuals, especially when dental records are complete [21].

Visualization of dental radiographs in clinical dental practice provides useful information for the most varied types of diagnosis and treatment plans. Through these radiographic records, in children and young adults, we can assess the development of dental maturity as they present morphologically different stages of formation and mineralization [7]. This is important in assessing and estimating an individual’s chronological age, for both clinical purposes and for medical-legal diagnosis.

Dental age can be determined by dental eruption and mineralization, the former being an event of short duration, related to the number of teeth emerging in the buccal cavity, during mixed dentition [22]. Besides being a rough process of estimating age (through dental eruption), teeth have been found to appear earlier in females than in males [14,23]. On the other hand, mineralization is a continuous, sequential, and uniform process [24,25,26], observable through radiographs, where various stages of tooth development (erupted and non-erupted) can be observed, from the formation of the crown and root, and ending with narrowing of the latter and closure of the radicular apex. The method of dental mineralization also presents differences between the [14,23,25] confirming that mineralization is also earlier in females than in males. According to Blenkin and Taylor [27], the mineralization process is very similar in both sexes until the age of the first menstruation, and from that time development is earlier in females.

Several methods have been proposed for estimating chronological age by means of dental maturity and mineralization, among which the Demirjian et al. [24] method is one of the most frequently used and tested across populations in order to verify its validity [3,5,18,19,28,29,30,31,32] and it has been considered the most suitable when compared with alternative proposals [31,33,34].

According to Demirjian et al. [24], to use dental age in different populations it is necessary to establish a standard through the populational samples studied, comparing the conversion table of the score of dental maturity for the dental age described in the method with the dental age of the population which is the subject of study.

The literature review tends to show that estimating chronological age by means of the dental mineralization stage is not a straightforward analysis as some contingent factors may influence the relationship, namely age group, sex, and the tooth under analysis. Several researchers found that the Demirjian standards need re-specification in order to correct for the over-estimation (e.g., Stamm et al. [35] for the population from Buenos Aires City; Moca et al. [36] for Romanian children). Staaf et al. [37] and Sobieska et al. [30] also found an overestimation of the Demirjian standards, the same occurring with a sample of Chinese children [38]. These limitations are not surprising considering that the population targeted in Demirjian’s study [24] was assumedly French-Canadians with French-Canadian ancestors. In this paper, we intend to test and highlight the applicability of the Demirjian method and contingent factors in the accuracy of estimating chronological age from dental maturity, through the following hypothesis: H1: the estimation of chronological age by means of the Demirjian scores varies across age groups both for global score and tooth score per se; and H2: the accuracy of estimation of chronological age by means of the Demirjian scores varies with sex, both for global score and tooth score per se.

## 2. Materials and Methods

### 2.1. Sample

The collected sample represents central and northern Portugal and Spain (Galicia). The sample comprises 574 dental radiographs from Portuguese (*n* = 270) and Spanish (*n* = 304) Caucasian children aged between 6 and 14 years, 296 boys (*M* = 10.42 years; *SD* = 2.42 years) and 278 girls (*M* = 10.27 years; *SD* = 2.33 years). The sample calculation was performed using the sample size determination formula based on the estimated proportion of the population with *p* (proportion of the population of individuals belonging to the category we are interested in studying) and *q* (proportion of the population of individuals not belonging to the category we are interested in studying, *1 − p*) unknown [39]. With a confidence level of 90% and α = 0.05, we would need at least 271 (rounded up from the figure of 270.6) subjects. We previously checked there are no differences between Portuguese and Spanish samples. Both populations are very similar (belonging to the Iberian Peninsula), and data were collected near the frontier between the two countries, for convenience reasons. No differences were found and we decided to present the results together.

### 2.2. Demirjian Method for Age Estimation

The Demirjian et al. [24] method uses analysis of the seven teeth present in the left mandibular hemiarcade, from the incisor to the second molar, from the dental radiographs of 1446 boys and 1482 girls aged between 2 and 20, of French-Canadian origin over two generations. Each tooth is classified according to a stage of maturity, according to diagrams with drawings of the teeth at each stage, establishing eight stages of mineralization designated by letters (A to H, qualitative analysis), from mineralization of the crown until the closure of the apex: each stage is attributed a score, with one table for females and another for males. In the technique described, all the values attributed to the total number of teeth of each individual are summed, this sum corresponding to the value of dental maturity, one for females and another for males on a scale from 0 to 100, using the same mathematical technique used by Tanner et al. [40], for skeletal age, this score corresponding to dental age. This total is converted in dental age using a table for converting the results of dental maturity [24].

### 2.3. Ethics, Procedures, and Data Analysis

The present study was part of research approved by the Faculty of Medicine at the University of Coimbra for Portuguese participants. All panoramic radiographs of Spanish participants are included in the database of personal information called “File#20: Patient management and clinical records of oral health” (School of Medicine and Dentistry, University of Santiago de Compostela). Informed consent was requested from the children’s parents/guardians.

Inclusion criteria: Children and adolescents aged 6 to 14 years. The following radiological criteria of exclusion were applied: lack of clarity of dental structures due to problems of contrast, movement, or artifacts; impacted teeth; radiopaque obturations or crowns; periapical lesions; endodontic treatment teeth; crowns bridging neighboring teeth.

To replicate Demirjian’s original procedure, we focused on 7 teeth from the left lower quadrant (from the central incisor to the second molar) to assess a subject’s dental maturity.

Data analyses were performed for each tooth and targeted age group and sex sub-samples using the Statistical Package for Social Sciences (IBM SPSS).

The radiographs were evaluated by two different examiners, both with experience in dental radiology. In order to test for inter-rater reliability, we used the intra-class correlation coefficient (ICC1) by Shrout and Fleiss [41]. Additionally, the test-retest was performed on categorizations (using Spearman’s correlation coefficient) made by the first author on two separate occasions (with a 6-month time lag).

Subjectivity plays an important role in categorizing dental maturity with the Demirjian standards. Some authors studied the accuracy assessment of dental age estimation with the Willems, Demirjian and Nolla methods in Spanish children [42]. Therefore, in order to control for subjectivity or possible bias created by the judge, we selected a stratified subsample of 72 individuals, representative of the larger sample. The first author categorized the dental radiographs following the Demirjian standards and we asked four more colleagues to categorize them independently. The rationale behind this procedure is that when subjectivity plays an insignificant role in making judgments, multiple judges will converge as to the categorization of the same objects. Considering the nature of the data and the number of observers, we used the Intraclass Correlation Coefficient (ICC1) as a suitable indicator of agreement [41]. ICC1 implies that we will use the original values of the first observer (the first author) in further analyses if the judgment is considered reliable.

Simple and multiple linear regressions were used to obtain a parsimonious model, allowing estimation of chronological age from the measurements made of the seven teeth based on each method and the variables of sex and age group, since in previous studies the Demirjian method is shown to be sensitive to these variables [7,30,35,36,37,38]. Using regression equations provides a more precise approach to chronological age based on Demirjian’s staging method [43]. The statistical assumptions of the models were analyzed and fulfilled, namely those of normal distribution, homogeneity, and independence of errors. The first two assumptions were validated graphically and the independence assumption was assessed with the Durbin–Watson statistic (values obtained close to 2) [44]. In the multiple regressions, we used the VIF coefficients to diagnose multicollinearity and no variable showed VIF indicators of multicollinearity (all VIF < 10). For all analyses, we considered a probability of type I error (α) = 0.05. Regression analysis builds upon the construction of equations that allows the prediction of the dependent variable (in this case chronological age) by means of explained variance of the Demirjian scores.

## 3. Results

### 3.1. Inter-Rater Reliability

The inter-rater agreement test of 72 individuals by means of the Intra-class Correlation Coefficient (ICC1), returned values indicating that subjectivity in codifying dental maturity is not a matter of concern in this study. ICC1 values found for each tooth ranged from 0.77 to 0.86 for single rater and 0.93 to 0.98 for the average of raters. ICC1 for Demirjian score (seven teeth) is even better with 0.92 for single rater and 0.98 for the average of raters thus showing clear convergence on decisions made.

Test-retest of ratings (6-month time lag) returned values ranging from 0.78 to 0.95 (all significant for *p* < 0.05), thus showing good consistency over time. Subjectivity should therefore be ruled out as a possible bias in later results, as shown in Table 1.

### 3.2. Chronological Age Forecast from the Global Score and Tooth by Tooth

When we took the sum of the seven teeth as the predictor and chronological age as a dependent variable, the analysis of the simple regression was found to be statistically significant (see Table 2). We obtained a predictive capacity of 65.1% of the total variance of the chronological age of participants using the Demirjian global score and of 71.7% of the total variance using the seven teeth (estimation in years). Setting out from the sample used and given the low rate of error associated with the inferential method used (under 1 possibility in 1000), the results showed we can apply the equation to forecast chronological age through the Demirjian global score with Portuguese and Spanish children, in order to forecast their respective chronological ages (see formulas for forecasting chronological age in the last lines of Table 2). Substituting the Demirjian scores in the equation with the values measured in each child, we obtain an estimate of chronological age.

Regression analyses differentiated by sex are shown in Table 3. The method was found to predict 68.7% of the total variance of chronological age for boys and 66.2% of the total variance for girls using the Demirjian global score and of 74.8% for boys and 75.0% for girls using the seven teeth. Therefore, and considering the effect size of the regression coefficients, we found that the Demirjian method is able to explain a greater proportion of total variance in boys and girls.

A comparison of the two sexes indicated that the method has a slight increase in predictive capacity for boys when we use the global score (2.5% more than for girls). However, when we use the seven teeth, the proportion of age estimation is very similar in boys and girls (74.8% of total variance for boys and 75.0% for girls). The equations forecasting chronological age for boys and girls from the Demirjian global score and for significant teeth are shown at the end of Table 3.

### 3.3. Predictive Capacities According to Age Group

Table 4 presents the comparison between children’s real chronological age and age estimated from the Demirjian score. As can be observed, there are differences in all age groups, for both boys and girls. The estimated marginal means of chronological age are also different, as shown in Table 3.

A comparison of children’s real chronological age and estimated age indicated, for both boys and girls, an overestimation when applying the Demirjian method. That overestimation is found in girls in all age groups, with higher values from 6 to 7 years and from 11 to 13 (see error column for girls in Table 3). For boys, overestimation is also found in all age groups except in 11- to 12-year-olds, where underestimation of chronological age (of −0.14 months) was found. On average, there was an overestimation of chronological age for both boys and girls, albeit greater in girls (average error of 15.13 months compared to 10.22 months in boys).

Table 5 presents the results of the regression analysis with the seven teeth assessed by the Demirjian method with a view to the estimation of each age group for boys and girls (we excluded 10 boys and 14 girls due to being aged between 14.01 and 14.40 years). As observed in Table 5, the predictive capacities of the Demirjian method and the significant teeth vary greatly as a function of children’s age. For boys, the global Demirjian score is only significant from 7 to 9 years and from 13 to 14. For girls, the method is able to predict ages from 6 to 10 years and from 11 to 12. From 12 years onwards, the Demirjian method loses predictive capacity in girls when considering the significance level *p* < 0.05. The youngest age groups (up to 9 years of age) are predicted most significantly. More specifically, the most strongly predicted age groups were identified in boys from 7 to 8 years of age (*R*^2^ = 76.9% of total variance) and from 8 to 9 years (*R*^2^ = 73.1%), and in girls from 6 to 7 years (*R*^2^ = 68.3%) and from 8 to 9 years (*R*^2^ = 64.1%). The age group least predicted by the method was from 10 to 11 years, for both boys (*R*^2^ = 20.7%) and girls (*R*^2^ = 21.3%).

### 3.4. Chronological Age Forecast from Tooth-by-Tooth Analysis

Finally, the regression equations of each tooth in boys and girls are presented (see Table 6). The R^2^ coefficient lets us compare the magnitude of the predictive capacities of each tooth. The most significant teeth are found to be the canine, second molar, and second pre-molar. The tooth with the least predictive capacity is the central incisor. A comparison of tooth-by-tooth estimation between boys and girls shows that the central incisor, lateral incisor, second pre-molar, and second molar have the greater predictive capacity in boys. In turn, the canine, first pre-molar, and first molar have the greater predictive capacity in girls.

## 4. Discussion

Estimation of children’s chronological age is important for several reasons and the Demirjian standards have been reported as accurate for this purpose. The Demirjian global score was able to predict 65.1% of the variance of the chronological age of Portuguese and Spanish children and using the seven teeth increases the predictive abilities to 71.7%. The Demirjian standards overestimated chronological age for both sexes, mainly for females. The predictive capacities of the Demirjian method and the significant teeth varied as a function of children’s age. Despite these results, there is an indication that the Demirjian scores are of limited use [7]. Koshy and Tandon [45] report a case in which Demirjian scores could not accurately predict the chronological age of 184 South Indian children. Australian researchers from Adelaide University found, in a sample of 655 South Australian children, that the Demirjian scores are of limited accuracy both for Australian-born and non-Australian-born children [46]. Tompkins [47] compared a sample of Caucasian French-Canadians with Black South African and Amerindian records and found substantial differences attributable to developmental patterns. Khorate and collaborators [48] used 500 panoramic radiographs of subjects from 4 to 22 years old in the state of Goa in India, applying the formulas of Chaillet, Demirjian et al. [24] modified [28]; the methods of Demirjian et al. [24] and Chaillet showed underestimation and inaccuracy of approximately 2 years. Tomás et al. [7] found that the accuracy of the Demirjian and Nolla methods varies across sex and age ranges: The Demirjian method tends to overestimate age and the Nolla method tends to underestimate it. These results are in line with those of other research studies [3,31,48,49].

Occasional reports of re-specification of the Demirjian standards in order to account for ethnic or age differences led us to hypothesize that there are contingency factors (age group, sex, and individual tooth development patterns) that may require further investigation.

The influential study of Demirjian et al. [24] has somehow integrated this idea by splitting the sample, taking into consideration the sex of individuals. Maturity development curves are explicitly different in boys and girls. We explored the same rationale and tested the accuracy of the Demirjian standards, splitting the sample into age groups (by means of clustering each tooth development stage simultaneously and looking for patterns). The results clearly show that the most strongly predicted age groups were identified in boys from 7 to 8 years old (*R*^2^ = 76.9%) and from 8 to 9 years (*R^2^* = 73.1%), while for girls it was 6 to 7 years old (*R*^2^ = 68.3%) and from 8 to 9 years (*R^2^* = 64.1%). The age group least predicted by the method was from 10 to 11 years, in both boys (*R*^2^ = 20.7%) and girls (*R*^2^ = 21.3%). Indeed, the literature shows that accuracy decreases with age even when using deciduous teeth for estimation [50]. Koshy and Tandon [45] also found that the accuracy of Demirjian standards varied according to age group, the authors finding that Demirjian standards are better for estimating young age groups.

Our results gave support to the two hypotheses formulated: H1: the estimation of chronological age by means of Demirjian scores varies across age groups both for global score and tooth score per se; and H2: The accuracy of estimation of chronological age by means of the Demirjian scores varies with sex, both for global score and tooth score per se.

Comparison between children’s real chronological age and estimated age indicated overestimation of chronological age for both boys (overestimation of 10.22 months) and girls (overestimation of 15.13 months). The application of the London atlas of tooth development and eruption showed an average overestimation of age by only one month in the Portuguese population, although further studies are needed [51]. Applying the Demirjian method to samples of different nationalities has largely led to authors concluding on overestimation of dental age, although some studies have indicated underestimation [31,35,52]. Among the studies finding underestimation is that of Cruz-Ladeira et al. [53] with Venezuelan subjects and that of Chen et al. [54] with Chinese individuals, although underestimation only occurred in boys; this result agrees with the 11 to 12 age group in boys in this study, where the method pointed towards slight underestimation. Overestimation was also found in older studies [14,15,16,18,20,28,29,33,54,55,56,57,58,59].

Revisiting the study by Cruz-Ladeira et al. [53], which assessed the dental radiographs of 308 Venezuelan and Spanish children aged between 2 and 18, the results showed that despite the significant correlation between dental and chronological age, the Demirjian et al. [24] method overestimated ages only in the Spanish sample, a result that agrees with ours. Bagherpour and collaborators [13] also concluded that the Demirjian et al. [24] method overestimated the age of Iranian boys by 0.34 years and that of girls by 0.25 years. The study by Feijóo et al. [56] also indicated an overestimation of dental age in relation to chronological age in boys (by 0.87 years on average) and girls (0.55 years). Jayaraman et al. [18] performed a meta-analysis of 274 studies where the Demirjian method was used. The authors recorded average overestimation of chronological age in all the studies (0.60 years for boys and 0.65 years for girls), except in a Chinese sample of boys and a Venezuelan one with boys and girls. We, therefore, conclude that our results regarding overestimation with the Demirjian et al. [24] method are in agreement with the literature.

In summary, our results are in line with studies indicating the existence of inaccuracies in estimating chronological age using the Demirjian method. For estimation with each tooth taken individually, the results lead us to conclude that the most significant teeth are the canine, the second molar, and the second pre-molar, with the central incisor being the tooth with the least predictive capacity. A comparison of tooth-by-tooth estimation between boys and girls reveals that the greatest predictive capacities lie in different teeth for boys and girls. As limitations of this research, the lack of proper validation methodology (adequate independent testing samples or cross-validation) should be noted, resulting in caution in the interpretation of results. The scope for generalizing the results becomes restricted since the sample did not cover all of the Iberian Peninsula. A larger sample, stratified according to age and sex, would allow a broader range of results, improving the external validity of the results.

## 5. Conclusions

The accuracy of estimating chronological age via the Demirjian standards is not as straightforward as it might appear, judging from the results found according to age group and sex crossed with age group. Research should develop in the direction of verifying which other factors should be included in order to gain a better understanding of the limits of chronological age estimation with the Demirjian standards.

It is also recommended when using the Demirjian standards that raters take special caution when the estimated age falls between 10 and 11 years for boys and girls, and in girls beyond 12 years of age. Notwithstanding these caveats, the Demirjian scores have predicted the chronological ages of individuals with an acceptable degree of accuracy. The only concern is that the degree of prediction is not as high as it may appear to be when the entire population is treated as a homogeneous group, especially when compared with segmentation by age and sex groups.

## Figures and Tables

**Table 1:** Inter-rater reliability.

		ICC (1) Consistency						
			95% Confidence Interval			95% Confidence Interval		
	Demirjian score	Single rater	Lower bound	Upper bound	Average of raters	Lower bound	Upper bound	*F* Value
ICC1	I1	0.82	0.749	0.870	0.95	0.923	0.964	18.633 ***
	I2	0.84	0.785	0.891	0.96	0.936	0.970	22.483 ***
	C	0.77	0.694	0.838	0.93	0.901	0.954	14.512 ***
	PM1	0.78	0.707	0.845	0.94	0.906	0.956	15.315 ***
	PM2	0.82	0.758	0.875	0.95	0.926	0.966	19.438 ***
	M1	0.82	0.756	0.875	0.95	0.926	0.965	19.360 ***
	M2	0.86	0.802	0.900	0.96	0.942	0.973	24.706 ***
	Global	0.92	0.892	0.947	0.98	0.971	0.986	48.762 ***

*** *p* < 0.001.

**Table 2:** Simple regression analysis of chronological age predicted by the Demirjian global score and tooth by tooth (*n* = 574).

Predictors:	*B*	*SE B*	*β*	*t*
(Constant)	−168.01	11.80		−14.24 ***
Demirjian global score	3.45	0.13	0.69	27.22 ***
*R* = 0.807, *R*^2^ = 0.651, *SEE* = 1.40, *F*(1, 572) = 1068.33, *p* < 0.001				
	*B*	*SE B*	*β*	*t*
(Constant)	−7.10	0.52		−13.67 ***
Lateral Incisor	−0.03	0.04	−0.03	−0.72
Central Incisor	−0.04	0.06	−0.03	−0.65
Canine	0.28	0.08	0.17	3.63 ***
1st Premolar	0.46	0.05	0.35	8.84 ***
2nd Premolar	0.06	0.07	0.05	0.94
1st Molar	0.22	0.03	0.22	7.25 ***
2nd Molar	0.40	0.05	0.34	7.99 ***
*R* = 0.847, *R*^2^ *=* 0.717, *SEE* = 1.27, *F*(1, 566) = 205.21, *p* < 0.001				
Equation forecasting chronological age through the Demirjian global score:				
Predicted chron. age = −168.01 + 3.45 × Demirjian score				
Equations forecasting chronological age through the 4 significant teeth:				
Predicted chron. age = −7.10 + 0.28 × Canine + 0.46 × 1st Premolar + 0.22 × 1st molar + 0.40 × 2nd Molar				

*** *p* < 0.001.

**Table 3:** Simple regression analysis of chronological age predicted by the Demirjian global score and tooth by tooth for boys (*n* = 296) girls and girls (*n* = 278).

Predictors:	Boys				Girls			
	*B*	*SE B*	*β*	*t*	*B*	*SE B*	*β*	*t*
(Constant)	−6.06	0.65		−9.27 ***	−8.24	0.80		−10.29 ***
Global score	0.19	0.01	0.83	25.38 ***	0.20	0.01	0.81	23.24 ***
*R* = 0.829, R^2^ = 0.687, *SEE* = 1.36, *F*(1, 294) = 644.12, *p* < 0.001					*R* = 0.814, R^2^ = 0.662, *SEE* = 1.36 *F*(1, 276) = 540.18, *p* < 0.001			
	*B*	*SE B*	*β*	*t*	*B*	*SE B*	*β*	*t*
(Constant)	−5.20	0.69		−7.58 ***	−7.86	0.76		−10.39 ***
Lateral Incisor	−0.07	0.06	−0.07	−1.21	0.06	0.06	0.06	1.00
Central Incisor	0.11	0.07	0.10	1.55	−0.01	0.09	−0.01	−0.17
Canine	0.24	0.10	0.14	2.32 *	0.44	0.11	0.26	3.94 ***
1st Premolar	0.37	0.07	0.31	5.13 ***	0.45	0.08	0.29	5.56 ***
2nd Premolar	0.18	0.09	0.13	1.90	0.13	0.10	0.09	1.38
1st Molar	−0.01	0.05	−0.01	−0.11	−0.14	0.08	−0.11	−1.83
2nd Molar	0.50	0.06	0.42	7.73 ***	0.55	0.08	0.44	6.66 ***
*R* = 0.865, *R*^2^ = 0.748, *SEE* = 1.23 *F*(1, 288) = 122.28, *p* < 0.001					*R* = 0.866, *R*^2^ = 0.750, *SEE* = 1.18 *F*(1, 270) = 115.93, *p* < 0.001			
Equations forecasting chronological age (estimation in years—global score):								
Predicted chronological age (boys) = −6.06 + 0.19 × Demirjian score					Predicted chronological age (girls) = −8.24 + 0.20 × Demirjian score			
Equations forecasting chronological age (estimation in years) through the 3 significant teeth:								
Predicted chronological age (boys) = −5.20 + 0.24 × Canine + 0.37 × 1st Premolar + 0.50 × 2nd Molar					Predicted chronological age (girls) = −7.86 + 0.44 × Canine + 0.45 × 1st Premolar + 0.55 × 2nd Molar			

* *p* < 0.05 *** *p* < 0.001.

**Table 4:** Λ Wilks and estimated marginal means of age (chronological and estimated by the Demirjian scores) for boys and girls according to age group.

Chronological Age	Λ Wilks		Age (Estimated Marginal Means—in Months)					
	Boys	Girls	Boys				Gilrs	
			Chronological (C)	Demirjian (D)	Error (D-C)	Chronological (C)	Demirjian (D)	Error (D-C)
6–7 years (72–83 months, *n =* 41)	0.03 *** (*n =* 21)	0.06 *** (*n =* 20)	72.39	90.81	18.42	72.19	91.81	19.62
7–8 years (84–95 months, *n =* 64)	0.29 *** (*n =* 29)	0.16 *** (*n =* 35)	88.83	102.31	13.48	87.86	98.86	11.00
8–9 years (96–107 months, *n =* 60)	0.16 *** (*n =* 36)	0.23 *** (*n =* 24)	98.94	107.11	8.17	102.25	114.88	12.63
9–10 years (108–119 months, *n =* 92)	0.19 *** (*n =* 42)	0.15 *** (*n =* 50)	113.52	124.38	10.86	115.08	127.22	12.14
10–11 years (120–131 months, *n =* 81)	0.22 *** (*n =* 37)	0.14 *** (*n =* 44)	124.46	133.11	8.65	125.36	146.23	20.87
11–12 years (132–143 months, *n =* 59)	0.23 *** (*n =* 29)	0.10 *** (*n =* 30)	137.97	137.83	−0.14	138.47	155.90	17.43
12–13 years (144–155 months, *n =* 49)	0.40 *** (*n =* 28)	0.06 *** (*n =* 21)	148.64	159.46	10.82	149.91	165.95	16.04
13–14 years (156–167 months, *n =* 101)	0.29 *** (*n =* 61)	0.20 *** (*n =* 40)	160.90	172.39	11.49	161.45	172.75	11.30
Global (mean)			118.21	128.43	10.22	117.07	134.20	15.13

*** *p* < 0.001. Note: 5 participants aged between 5.5 and 5.9 years old and 27 participants aged between 14 and 14.4 years old were excluded from this analysis.

**Table 5:** Regression analysis for boys and girls according to age group (estimation in years; regression equations for significant teeth, *p* < 0.05).

Chronological Age	Boys	Girls
6–7 years (72–83 months)	*R* = 0.713, *R*^2^ = 0.509, *SEE* = 0.23 *F*(7, 16) = 2.37, *p* = 0 0.073 [*n* = 24]	*R =* 0.827, *R*^2^ = 0.683, *SEE* = 0.21 *F*(7, 12) = 3.70, *p* = 0.023 [*n* = 20]
		Chronological age = 5.01 + 0.22 × canine
7–8 years (84–95 months)	*R* = 0.877, R^2^ = 0.769, SEE = 0.17 *F*(7, 21) = 9.97, *p* < 0.001 [*n* = 29]	*R* = 0.759, *R*^2^ = 0.577, *SEE* = 0.22 *F*(7,27) = 5.25, *p* = 0.001 [*n* = 35]
	Chronological age = 7.03 − 0.09 × 1st molar + 0.072nd molar – 0.08 × central incisor + 0.08 × lateral incisor + 0.06 1st premolar	Chronological age = 2.72 − 0.15 × 2nd premolar
8–9 years (96–107 months)	*R* = 0.855, R^2^ = 0.731, *SEE* = 0.17 *F*(7, 28) = 10.85, *p* < 0.001 [n = 36]	*R* = 0.800, R^2^ = 0.641, *SEE* = 0.23 *F*(7, 16) = 4.08, *p* = 0.009 [*n* = 24]
	Chronological age = 5.37 + 0.13 × 2nd molar + 0.04 × central incisor	Chronological age = 4.65+ 0.22 × 2nd premolar
9–10 years (108–119 months)	*R* = 0.529, *R*^2^ = 0.280, *SEE* = 0.25 *F*(7, 34) =1.89, *p* = 0.102 [*n* = 42]	*R* = 0.633, *R*^2^ = 0.401, *SEE* = 0.21 *F*(7, 42) = 4.02, *p* = 0.002 [*n* = 50]
	Chronological age = 8.06 + 0.09 × lateral incisor	Chronological age = 7.02 + 0.11 × canine + 0.10 × central incisor − 0.131st premolar
10–11 years (120–131 months)	*R* = 0.455, *R*^2^ = 0.207, *SEE* = 0.33 *F*(7, 29) = 1.08, *p* = 0.399 [*n* = 37]	*R* = 0.462, *R*^2^ = 0.213, *SEE* = 0.28 *F*(7, 36) = 1.39, *p* = 0.238 [*n* = 44]
11–12 years (132–143 months)	*R* = 0.623, *R*^2^ = 0.388, *SEE* = 0.24 *F*(7, 21) = 1.90, *p* = 0.121 [*n* = 29]	*R* = 0.700, *R*^2^ = 0.491, *SEE* = 0.24 *F*(7, 22) = 3.03, *p* = 0.022 [*n* = 30]
		Chronological age = −0.75 + 0.28 × 2nd molar + 0.17 × lateral incisor
12–13 years (144–155 months)	*R* = 0.655, *R*^2^ = 0.429, *SEE* = 0.21 *F*(6, 21) = 2.63, *p* = 0.047 [n = 28]	*R* = 0.645, *R*^2^ = 0.416, *SEE* = 0.23 *F*(5, 15) = 2.14, *p =* 0.116 [*n* = 21]
	Chronological age = 21.99 + 0.26 × 2nd molar + 0.60 × 1st molar − 0.42 × 1st premolar	
13–14 years (156–167 months)	*R* = 0.519, *R*^2^ = 0.269, *SEE* = 0.23 *F*(6, 54) = 3.32, *p* = 0.007 [n = 61]	*R* = 0.467, *R*^2^ = 0.218, *SEE* = 0.24 *F*(5, 34) = 1.90, *p* = 0.120 [*n* = 40]
	Chronological age = 11.51 + 0.21 × 2nd molar + 0.37 × central incisor − 0.44 × 1st premolar	

**Table 6:** Regression equations of each tooth individually for boys and girls (estimation in years).

Tooth	Boys	Girls
Central incisor	Chronological age = 4.95 + 0.43 × Central incisor	Chronological age = 5.14 + 0.37 × Central incisor
	*R* = 0.402, *R*^2^ = 0.161, *SEE* = 2.22 *F*(1, 294) = 56.62, *p* < 0.001	*R* = 0.348, *R*^2^ = 0.121, *SEE* = 2.19 *F*(1, 276) = 38.04, *p* < 0.001
Lateral incisor	Chronological age = 0.99 + 0.78 × Lateral incisor	Chronological age = −0.46 + 0.82 × Lateral incisor
	*R* = 0.668, *R*^2^ = 0.446, *SEE* = 1.80 *F*(1, 294) = 236.49, *p* < 0.001	*R* = 0.615, *R*^2^ = 0.378, *SEE* = 1.84 *F*(1, 276) = 167.79, *p* < 0.001
Canine	Chronological age = −2.79 + 1.27 × canine	Chronological age = −4.81 + 1.34 × canine
	*R* = 0.767, *R*^2^ = 0.588, *SEE* = 1.56 *F*(1, 294) = 419.04, *p* < 0.001	*R* = 0.797, *R*^2^ = 0.635, *SEE* = 1.41 *F*(1, 276) = 480.00, *p* < 0.001
First pre-molar	Chronological age = 1.13 + 0.70 × first pre-molar	Chronological age = −4.14 + 10.06 × first pre-molar
	*R* = 0.583, *R*^2^ = 0.340, *SEE* = 1.97 *F*(1, 294) = 151.35, *p* < 0.001	*R* = 0.687, *R*^2^ = 0.472, *SEE* = 1.70 *F*(1, 276) = 246.87, *p* < 0.001
Second pre-molar	Chronological age = −1.73 + 1.03 × second pre-molar	Chronological age = −2.67 + 1.03 × second pre-molar
	*R* = 0.764, *R*^2^ = 0.583, *SEE* = 1.56 *F*(1, 294) = 411.12, *p* < 0.001	*R* = 0.711, *R*^2^ = 0.506, *SEE* = 1.64 *F*(1, 276) = 282.49, *p* < 0.001
First molar	Chronological age = 1.35 + 0.54 × first molar	Chronological age = −2.72 + 0.87 × first molar
	*R* = 0.561, *R*^2^ = 0.315, *SEE* = 2.01 *F*(1, 294) = 134.95, *p* < 0.001	*R* = 0.645, *R*^2^ = 0.417, *SEE* = 1.78 *F*(1, 276) = 197.10, *p* < 0.001
Second molar	Chronological age = −0.30 +0.92 × second molar	Chronological age = −1.15 + 0.91 × second molar
	*R* = 0.774, *R*^2^ = 0.599, *SEE* = 1.53 *F*(1, 294) = 439.28, *p* < 0.001	*R* = 0.743, *R*^2^ = 0.553, *SEE* = 1.56 *F*(1, 276) = 340.31, *p* < 0.001

## Data Availability

The data presented in this study are available on request from the corresponding author.

## Abbreviations

B	Unstandardized regression coefficient
β	Standardized coefficient
C	Chronological
D	Demirjian
F	F-test
M	Mean
*p*	Significance level
R	Linear regression coefficient
R^2^	R Squared (coefficient of determination)
SD	Standard-deviation
SE B	Standard-error for unstandardized coefficient
SE	Standard-error
